# Antifungal effects of echinocandins diminish when exposed to intestinal lumen contents: a finding with potentially significant clinical implications

**DOI:** 10.3389/fphar.2024.1376656

**Published:** 2024-03-27

**Authors:** Sepideh Mehravar, Gabriela S. Leite, Mark Pimentel, Ali Rezaie

**Affiliations:** ^1^ Medically Associated Science and Technology (MAST) Program, Cedars Sinai Medical Center, Los Angeles, CA, United States; ^2^ Karsh Division of Gastroenterology and Hepatology, Department of Medicine, Cedars Sinai Medical Center, Los Angeles, CA, United States

**Keywords:** echinocandins, caspofungin, micafungin, fungal infection, gastrointestinal tract

## Abstract

Echinocandins, a prominent class of antifungals, are known for their broad-spectrum activity and favorable safety profiles. However, their bioavailability and efficacy via oral route are suboptimal. In this study, caspofungin and micafungin, the two most commonly used echinocandins, were evaluated in various *in vitro* environments simulating intestinal lumen. The results revealed that while both antifungals are effective in standard medium, their efficacy significantly diminishes in the presence of human small bowel aspirates and bovine bile. The study suggests that bowel contents and specifically bile acids may be a suppressive component, hindering the antifungal effects of echinocandins. This novel exploration sheds light on the poor oral bioavailability of echinocandins. The findings imply that echinocandins alone, regardless of administration route, may not be optimal for gastrointestinal (GI) fungal infections or invasive fungal infections originating from intestinal translocation. Further clinical investigations are warranted to validate and expand upon these observations.

## Introduction

Fungal infections increasingly pose a global health challenge particularly in developing countries. Over the past 80 years, development of antifungal medications has seen significant advancements along with ongoing hurdles including effectiveness, side effects, access, and rise of antifungal resistance.

Echinocandins are a major class of broad-spectrum antifungals (e.g., caspofungin and micafungin) with favorable safety profiles. They are characterized as large lipopeptide molecules and act as inhibitors of β-(1,3)-glucan synthesis, causing damage to fungal cell walls. They are commonly used in invasive candidiasis and aspergillosis along with empiric antifungal therapy in neutropenic fever and prophylaxis against invasive fungal infections (Perlin, 2011).

Echinocandins have common traits as amphiphilic cyclic hexapeptides, featuring an N-linked acyl lipid sidechain and an approximate molecular weight of 1200. Caspofungin (acetate) exhibits high solubility in both water and methanol, while micafungin (sodium) is freely soluble in water. Notably, echinocandins are believed to lack sufficient bioavailability for oral administration, with less than 0.2% bioavailability noted for caspofungin (Kurtz and Rex, 2001). In a clinical trial involving patients with AIDS, oral administration of anidulafungin at doses up to 500 mg per day resulted in a low peak plasma concentrations of 753 ng/mL and was found to be inadequate to consistently achieve favorable outcomes against oropharyngeal candidiasis (Denning, 2003).

It is not clear if low oral bioavailability of echinocandins is due to breakdown in the gastrointestinal (GI) tract or simply lack of GI absorbability; however, the distinction has significant clinical implications. If echinocandins are neutralized in the GI tract, they may not be an optimal choice for GI fungal infections or invasive fungal infections with a GI source. Hence, we aimed to assess the efficacy of caspofungin and micafungin against *Candida albicans* in various GI luminal simulations *in vitro* including human duodenal aspirates and bovine bile.

## Materials and methods

### Organism and media

*C*. *albicans* (ATCC 10231) liquid cultures in Sabouraud (SBD) medium were obtained from −80°C stock solutions (preserved in Tris-buffered saline containing 25% glycerol and YPD broth), and sub-cultured onto SBD agar plates (Teknova), at 37°C for a duration of 24–48 h to obtain single colonies. A single colony was selected and sub-cultured at 37°C for 24 h prior testing with anti-fungi agents in combination with filtered bovine bile medium (B3883 Sigma) and endoscopically obtained human duodenal aspirate fluid.

After informed consent was obtained, subjects aged 18–85 years undergoing esophagogastroduodenoscopy (EGD) for standard of care purposes were recruited (Cedars-Sinai IRB 35192). Fresh duodenal aspirates were obtained during upper endoscopy (without colon preparation) using a validated dual-lumen sterile catheter system to avoid contamination with saliva. In a subset of experiments, aspirates were also filtered using a 0.22 µm filters or autoclaved before susceptibility test with antifungal agents.

### Antifungal agents

Caspofungin acetate (Fosun Pharma., NJ, USA) and Micafungin (Hikma Pharmaceuticals PLC, London) were dissolved in sterile distilled water to a final concentration of 5 mg/mL. Aliquots were stored in glass vials at −20°C, protected from light.

### Susceptibility and intestinal simulation experiments

A modified microtiter test (CLSI M27-A accepted standard (Wayne, 2002)) was used to check the minimal inhibitory concentration (MIC) of each drug in the presence of bovine bile and human duodenal aspirates. 96 well microtiter plates were prepared by serially diluting each agent along the x-axis to a final concentration ranging from 0.001 to 2 μg/mL in SBD medium (controls), 0.0002–2048 μg/mL in bovine bile (50 mg/mL) or 0.125–16 μg/mL in human duodenal aspirates (diluted 3x with sterile 1x PBS). Aspirates were obtained from 5 subjects undergoing standard of care esophagogastroduodenoscopy for upper GI symptoms. Patients provided consent and study is approved by the institutional review board at Cedars-Sinai Medical Center. Yeast suspensions were prepared by suspending single colonies in sterile 0.85% saline. The turbidity of each suspension was measured on a spectrophotometer at an optical density (OD) of 530 nm. Yeast suspensions were then adjusted using the appropriate medium (SBD, bovine bile, or human duodenal aspirates), to give a final inoculum of 2.5 × 10^3^ CFU/mL per well. One well of each plate was left agent-free to act as a positive control. Negative controls were also included to ensure the sterility of each medium. Plates were first incubated in a moist chamber at 37°C for 24–48 h, and then shaken for 1 min to obtain a uniform suspension prior checking the OD 530 nm (Biotek Synergy H1 microplate reader). The minimum inhibitory concentration (MIC) was taken as the lowest drug concentration that reduced the OD_530_ by 100% compared with the drug-free control. Experiments were done in triplicates. To eliminate the effect of gut microbiome, 0.22 μm sterile filtering (Whatman 9913-2502) and autoclave were used on duodenal aspirates. This was to assess whether gut microbiome has any neutralizing effect on echinocandins. Furthermore, bovine bile was used to assess if the effects of intestinal aspirates are potentially mediated by bile.

## Results

**FIGURE 1:**
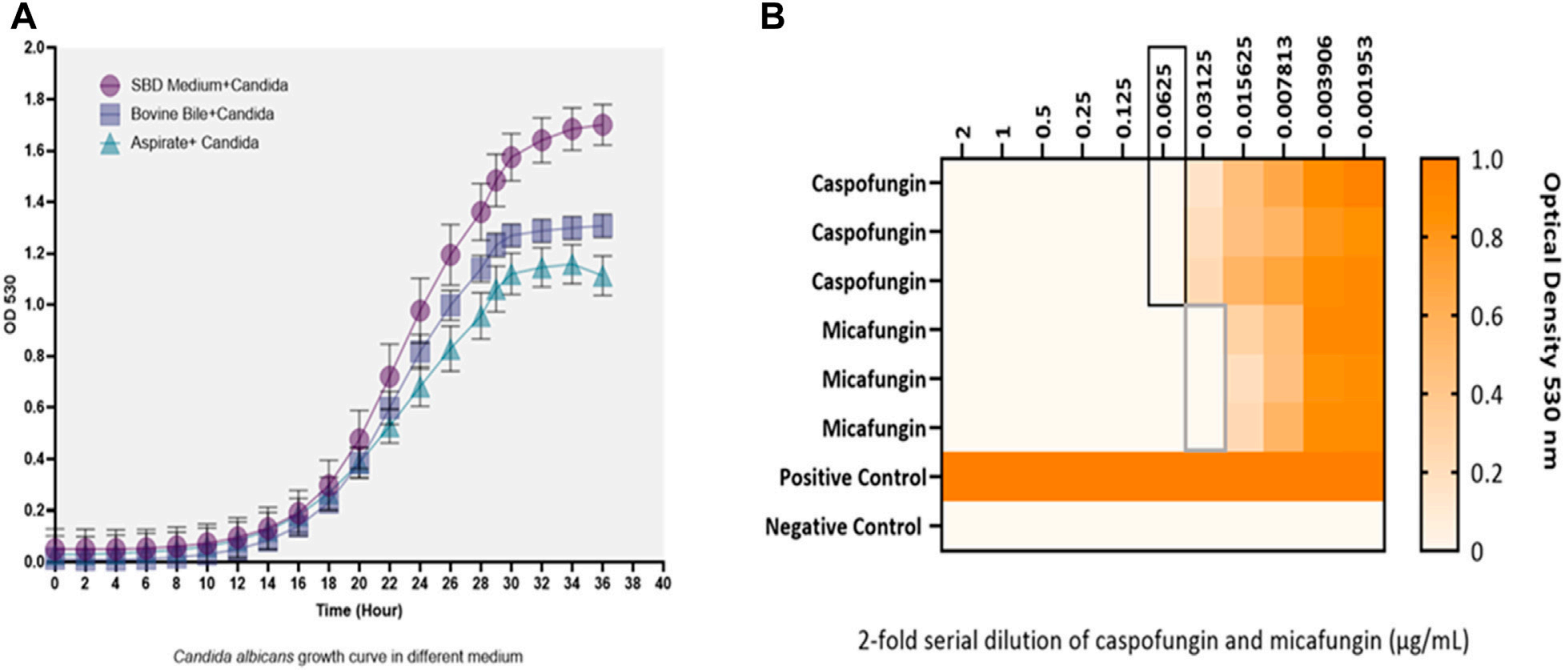
**(A)** Kinetic growth curves of *Candida* albicans in three different cultured mediums (SBD medium, bovine bile, and duodenal aspirate). Changes are shown based on optical density (OD) over time. **(B)** The minimum inhibitory concentration (MIC) of caspofungin and micafungin against *Candida albicans* in Sabouraud medium. Black and gray boxes show MIC for caspofungin and micafungin, respectively. *Candida albicans* with a final concentration of 2.5 × 10^3^ CFU/mL were exposed to different concentrations of caspofungin and micafungin (0.001–2 μg/mL), and the optical densities (OD530) were measured after 24–48 h. The heat map is based on the OD average of three individual experiments.

**FIGURE 2:**
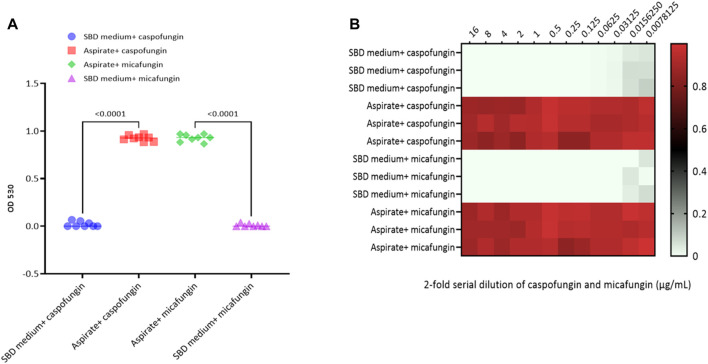
Candida albicans optical density (OD530) in the presence of **(A)** micafungin (0.03125 μg/ml) and caspofungin (0.0625 μg/ml) in SBD medium and human duodenal aspirates; **(B)** micafungin (0.0007 to 16 μg/ml) and caspofungin (0.0007 to 16 μg/ml) in human duodenal aspirates (diluted 3x) compared to SBD medium (controls). The ODs were measured after 24 to 48 hours. The heat map is based on the average of three individual experiments. The gradient of light green to red indicates the change in OD530 values (minimum to maximum).

**FIGURE 3:**
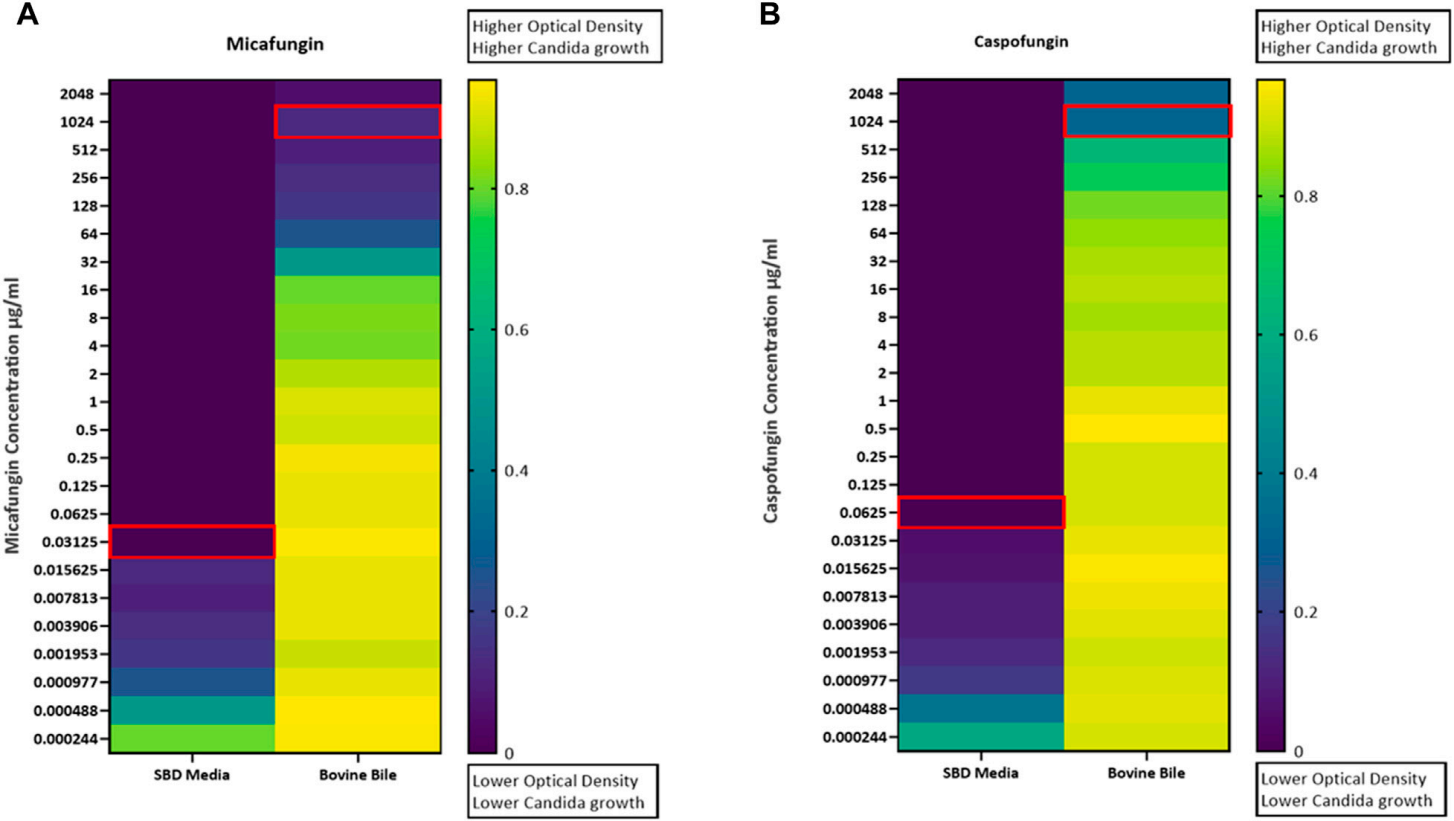
*Candida albicans* optical density (OD530) in the presence of **(A)** micafungin (0.0002–2048 μg/mL) and **(B)** caspofungin (0.0002–2048 μg/mL) and bovine bile (50 mg/mL) compared to SBD medium (controls). The ODs were measured after 24–48 h. The heat map is based on the average of three individual experiments. The gradient of dark blue to yellow indicates the change in OD530 values (minimum to maximum).

Kinetic growth curves of C. albicans in various cultured mediums (SBD medium, bovine bile, and duodenal aspirate) under normal, untreated growth conditions are shown in Figure 1A. The MICs for *C. albicans* in SBD medium for caspofungin and micafungin were 0.0625 μg/mL and 0.01325 μg/mL, respectively (Figure 1B). In the presence of human duodenal aspirates, caspofungin at 0.0625 μg/mL and micafungin at 0.01325 μg/mL exhibited no inhibitory effect against *C. albicans* when human duodenal aspirates were tested (Figure 2A). Furthermore, when exposed to duodenal aspirates, caspofungin and micafungin had no effect against *C. albicans*, even at high doses (16 µg/mL-0.126 μg/mL, Figure 2B). Caspofungin and micafungin remained ineffective when exposed to filtered and autoclaved aspirates suggesting that their inefficacy is not mediated by microbiome when compared to experiments performed with neat aspirates (data not shown). Similarly, the MIC of caspofungin and micafungin also had no effect regardless the dose tested (0.0002–2048 μg/mL) when bovine bile was used (Figure 3), suggesting that bile potentially is the suppressive component in aspirates regarding the antifungal effects of micafungin and caspofungin. Of note measured pH of the SBD medium, small bowel aspirates and bile were 6.0, 7.0, and 7.0, respectively.

## Discussion

In this study, we show that *C. albicans* is expectedly susceptible to caspofungin and micafungin in SBD medium; however, the presence of bovine bile and human duodenal aspirates dramatically reduce the antifungal effect of these agents, even at high concentrations. Further, inhibitory effect observed in duodenal aspirates may be attributed to bile acids, hindering the efficacy of echinocandins.

Our study is a novel exploration of underlying cause for known poor oral bioavailability of echinocandins and mechanisms behind their lack of efficacy when administered orally.

Our findings are in line and in part, explain the findings by Healy et al. In an elegant study, Healy et al. presented a *Candida* glabrata GI colonization mouse model to explore the effect of caspofungin on luminally colonized yeast. Within this study, the pharmacokinetics of intraperitoneally administered caspofungin were measured within the GI tract following a single-dose administration of 5 or 20 mg/kg. The 5 mg/kg dose is considered the equivalent humanized (therapeutic) dose. Significantly reduced luminal caspofungin drug levels were observed and levels were not maintained at a high level for a sufficiently long time (Healey et al., 2017). This observation is of utmost importance as the GI tract can serve as the main site of fungal colonization specially in the immunocompromised hosts. Reduced penetration of echinocandins into the GI lumen as shown by Healy et al. along with our finding of neutralization of echinocandins in the GI lumen can allow regrowth and even antifungal resistance acquisition of fungal cells inside the GI tract.

To our knowledge, there are no studies to evaluate the concentration of echinocandins in the intestinal lumen when administered intravenously; however, our findings suggest that echinocandins localizing to the bowel lumen, irrespective of the given route, do not exhibit antifungal effects. Hence, echinocandins may not be optimal choices for fungal infections originating from intestinal translocation. However, further clinical and *in-vivo* investigations are imperative to evaluate and expand upon our findings.

## Data Availability

The raw data supporting the conclusion of this article will be made available by the authors, without undue reservation.
